# MRI-based radiomics model for predicting tumor regression patterns after neoadjuvant chemotherapy in breast cancer

**DOI:** 10.3389/fmed.2025.1661448

**Published:** 2025-11-17

**Authors:** Lan Wang, Qi Wang, Jun Zhang, Meng Zhang, Tianhui Guo, Wen Gao, Biyuan Zhang, Haiji Wang

**Affiliations:** 1 Department of Radiation Oncology, Affiliated Hospital of Qingdao University, Qingdao, China; 2 Department of Oncology, Jinan Third People's Hospital, Jinan, China

**Keywords:** breast cancer, radiomics, MRI, neoadjuvant chemotherapy, tumor regression pattern

## Abstract

**Purpose:**

We investigated a predictive framework that integrates MRI-derived radiomic characteristics with clinical indicators to assess how breast tumors respond to neoadjuvant chemotherapy.

**Methods:**

A retrospective review was conducted on 301 patients with pathologically confirmed breast cancer. From their baseline MRI scans, 1,196 radiomic features were extracted. Feature reduction was carried out through ANOVA followed by LASSO regression to select the most relevant variables. Eight machine learning algorithms, including Random Forest and XGBoost, were used to develop predictive models incorporating both radiomic and clinical data. Patients were randomly divided into a training set (*n* = 240) and a validation set (*n* = 61). Model performance was assessed using the area under the ROC curve (AUC), sensitivity, specificity, and accuracy.

**Results:**

In performance evaluation, the Random Forest approach yielded area under the curve values of 0.82 for training and 0.75 for validation, reflecting consistent predictive strength. A nomogram constructed using the selected features achieved an AUC of 0.75 in the validation cohort, with a sensitivity of 0.64 and a specificity of 0.88.

**Conclusion:**

The integration of imaging biomarkers and clinical profiles enables reliable prediction of tumor response post-NAC, supporting more informed and tailored treatment strategies.

## 1 Introduction

Breast cancer remains one of the most prevalent malignancies across the globe and contributes substantially to cancer-related deaths among women (1). Neoadjuvant chemotherapy (NAC) is frequently employed in cases of locally advanced breast cancer to reduce tumor size and enhance the feasibility of breast-conserving surgery (BCS) (2, 3). However, responses to NAC vary greatly among patients because of tumor heterogeneity. Tumor shrinkage patterns after NAC are prognostically relevant and increasingly used to guide individualized treatment (4).

Differences in regression patterns after NAC strongly influence surgical decision-making (5). Tumor regression is commonly categorized as either concentric regression (CR) or non-concentric regression (NCR). A typical feature of CR is a consistent, inward pattern of shrinkage, often leaving behind a solitary residual lesion or achieving full pathological resolution. Such regression allows clearer tumor boundary identification, improving surgical outcomes. Research involving MDCT has shown BCS success rates reaching up to 94% (6). In contrast, NCR is often associated with irregular tumor shrinkage, fragmented residual foci, or a mesh-like appearance, complicating the evaluation of residual disease extent (6). The RCB system standardizes evaluation of tumor burden after NAC; an RCB-III score reflects heavy residual disease and greater recurrence risk (7, 8). Emerging evidence suggests that the immune context of the tumor microenvironment can affect the trajectory of tumor regression. Notably, an increased presence of tumor-infiltrating lymphocytes (TILs) has been linked to enhanced responsiveness to neoadjuvant chemotherapy (9). Thus, precise evaluation of regression patterns using imaging modalities is critical for guiding personalized treatment strategies.

MRI has become integral to breast cancer evaluation, as it captures high-resolution insights into tumor vascular structures and functional characteristics (10). Compared to traditional imaging methods, MRI offers enhanced sensitivity in detecting non-mass enhancement, delineating tumor edges, and monitoring morphological changes during therapy, making it valuable for pre-surgical evaluation (11). Nevertheless, MRI’s accuracy in identifying residual lesions post-NAC is sometimes compromised by misinterpretations—both false positives and negatives—which may hinder optimal surgical planning (12). This limitation partly stems from MRI’s reduced sensitivity to post-treatment histological changes like necrosis and fibrosis (13). In cases of NCR, irregular tumor cell dispersion and complex stromal architecture may mask enhancement signals, thereby raising the likelihood of diagnostic errors (14). Moreover, NCR-type tumors often exhibit poorly defined boundaries and trigger immune reactions that minimally impact perfusion, limiting the effectiveness of quantitative MRI metrics (15). Integrating radiomics features with clinical data presents a potential approach to bridge diagnostic limitations and refine MRI-based classification of regression types.

Radiomics refers to the process of extracting large-scale quantitative data from routine medical images, enabling non-invasive insights into tumor biological characteristics such as spatial heterogeneity and therapeutic response (16). Evidence from multiple centers indicates that when radiomic features are integrated with clinical indicators, they can support accurate prediction of recurrence-free survival (RFS) and overall survival (OS) in individuals with breast cancer (17). This research proposes a predictive model that integrates radiomic attributes derived from MRI with clinicopathological factors to classify tumor regression patterns following NAC, aiming to identify dependable imaging indicators for informing personalized treatment strategies.

## 2 Materials and methods

### 2.1 Patient population

A retrospective review was conducted on clinical and imaging records of 301 breast cancer patients who received treatment at the Affiliated Hospital of Qingdao University between September 2022 and September 2024. Inclusion and exclusion were determined based on standardized enrollment criteria. Individuals were eligible if they satisfied all of the following: (a) breast cancer confirmed by histopathology through core needle biopsy; (b) baseline breast MRI performed in-house prior to therapy; (c) complete clinical and pathological baseline records; and (d) definitive surgery following a standard NAC protocol. Participants were excluded if they had: (a) no MRI or low-quality imaging; (b) surgery conducted at other institutions without available postoperative pathology reports; or (c) other malignancies diagnosed concurrently during the study period.

The enrolled cases were randomly split into a training group (*n* = 240; CR: 182; NCR: 58) and a validation group (*n* = 61; CR: 45; NCR: 16) based on a 7:3 allocation ratio. All NAC regimens followed NCCN guidelines and were tailored through multidisciplinary team (MDT) evaluations. Treatment typically lasted 8 weeks (IQR: 6–8 weeks). Institutional ethics approval was granted by the Affiliated Hospital of Qingdao University (Approval No.: QYFY WZLL 27741). Due to the retrospective nature of this study, the requirement for informed consent was waived.

### 2.2 Tumor regression pattern classification

Post-treatment tumor regression subtypes were determined by histopathological analysis, in alignment with assessment frameworks established by the NCCN and Miller–Payne (MP) grading systems. Excised tissue was preserved in 10% neutral-buffered formalin, and independently reviewed by two certified pathologists under blinded conditions. Any disagreement in evaluation was adjudicated by a senior expert through consensus.

Based on the distribution and quantity of residual lesions post-NAC, patients were classified into CR or NCR categories. CR typically presents as a single, localized regression focus or as pathological complete response (pCR), defined by the absence of invasive tumor in both the breast and axillary lymph nodes. Residual ductal carcinoma *in situ* (DCIS) was not considered exclusionary. In contrast, NCR encompassed scenarios such as multifocal residual tumors, irregular regression patterns, central regression with peripheral nodules, and cases exhibiting either disease stability or progression.

For subsequent radiomics modeling, additional pathological features were extracted, including the maximal diameter of residual lesions (according to American Joint Committee on Cancer (AJCC) 8th edition), regression margin characteristics, and dynamic variations in ER, PR, and HER2 status.

### 2.3 MRI acquisition

Bilateral breast magnetic resonance imaging was carried out for each patient prior to NAC, using clinical-grade scanners operating at either 1.5 Tesla or 3.0 Tesla. Scans were performed with the patient in the prone position, utilizing a dedicated multi-channel breast coil to enhance spatial resolution and suppress motion artifacts. The imaging protocol included high-resolution T1- and T2-weighted sequences to provide detailed anatomical visualization of breast tissue. For contrast-enhanced imaging, dynamic scans were obtained using a fat-suppressed volumetric interpolated breath-hold examination (VIBE) technique, applied across multiple phases.

Delayed post-contrast sequences were also acquired to assess lesion morphology and contrast enhancement kinetics. Imaging parameters were standardized in accordance with international breast MRI protocols to support reproducibility and ensure data comparability for radiomic feature extraction.

### 2.4 Radiomic feature extraction and model construction

Tumor boundaries were manually delineated on baseline T1-weighted MRI scans, chosen for their superior contrast in outlining lesions. Delineation was independently carried out by two senior radiologists using ITK-SNAP software. Any disagreements were reviewed collaboratively to reach consensus, with inter-observer agreement exceeding a Kappa value of 0.75.

Each segmented region yielded a broad spectrum of radiomic features, such as geometric descriptors, grayscale statistics, and texture-based metrics. Derived variables were generated via mathematical transformations of the original set. Redundant features were filtered out using Pearson correlation matrices, followed by the LASSO method to retain high-value predictors.

Radiomic feature extraction and selection were implemented through PyRadiomics (v3.0.1). The refined features were fed into supervised classifiers developed using the scikit-learn library. Performance was evaluated on an independent dataset. Four machine learning methods—logistic regression (LR), support vector machine (SVM), random forest (RF), and extreme gradient boosting (XGBoost)—were tested for distinguishing CR and NCR patterns.

### 2.5 Clinical prediction model

A clinical classification framework was devised to distinguish between different tumor regression profiles after neoadjuvant chemotherapy. Input features were collected prior to treatment and included age, menopausal state, hormone receptor (ER/PR) expression, Ki-67 index, HER2 amplification (confirmed by FISH), clinical tumor size (cT), nodal involvement (cN), and pathological response indicators.

The Miller–Payne (MP) grading method was employed to evaluate histologic changes in cellularity by comparing tumor tissues before and after NAC. This five-grade scale accounts for a continuum of responses, from minimal residual disease to complete clearance of invasive malignancy. In the training dataset, univariate testing identified significant predictors, and those with *p*-values below 0.1 were retained. The final subset of predictors included four variables: age, ER status, PR status, and cN stage.

Predictive models were generated using four machine learning techniques: logistic regression (LR), support vector machine (SVM), random forest (RF), and extreme gradient boosting (XGBoost). A stratified 10-fold cross-validation was performed to evaluate model performance. The dataset was randomly divided into 10 folds, ensuring similar CR and NCR case ratios in each. In every iteration, nine folds were used for training and one for validation, and the process was repeated 10 times. The average performance across folds was reported as the final result.

Model performance was assessed using accuracy, sensitivity, specificity, and AUC. Hyperparameters were optimized by grid search during cross-validation. The Random Forest and XGBoost models were tuned accordingly, and the model with the highest mean AUC was selected for nomogram development.

### 2.6 Nomogram construction

To enhance individualized clinical decision-making, a hybrid prediction model was developed by integrating radiomic signatures from pre-treatment MRI with key baseline clinical parameters. A nomogram was constructed based on this combined model to visualize patient-specific probabilities of tumor regression types. To confirm the model’s robustness, its performance was independently validated using an external patient dataset.

### 2.7 Statistical analysis

Statistical analyses were conducted using Python (version 3.7) and R software (version 4.3.0). Continuous variables were analyzed with two-sample t-tests, while categorical data were compared using chi-square statistics. Feature filtering involved two stages: univariate t-tests (*p* < 0.05) for initial selection, followed by removal of highly correlated variables using Pearson correlation thresholds (r > 0.9), keeping one representative per correlation group.

LASSO regularization, performed via the “glmnet” package in R, was applied to finalize variable selection and assist model construction. Predictive capability was quantified using AUC values derived from ROC curves. Additional indicators such as sensitivity, specificity, and accuracy were employed to evaluate overall classification quality. A two-sided *p*-value < 0.05 was set to indicate statistical significance.

## 3 Results

### 3.1 Patient characteristics

**Figure 1:**
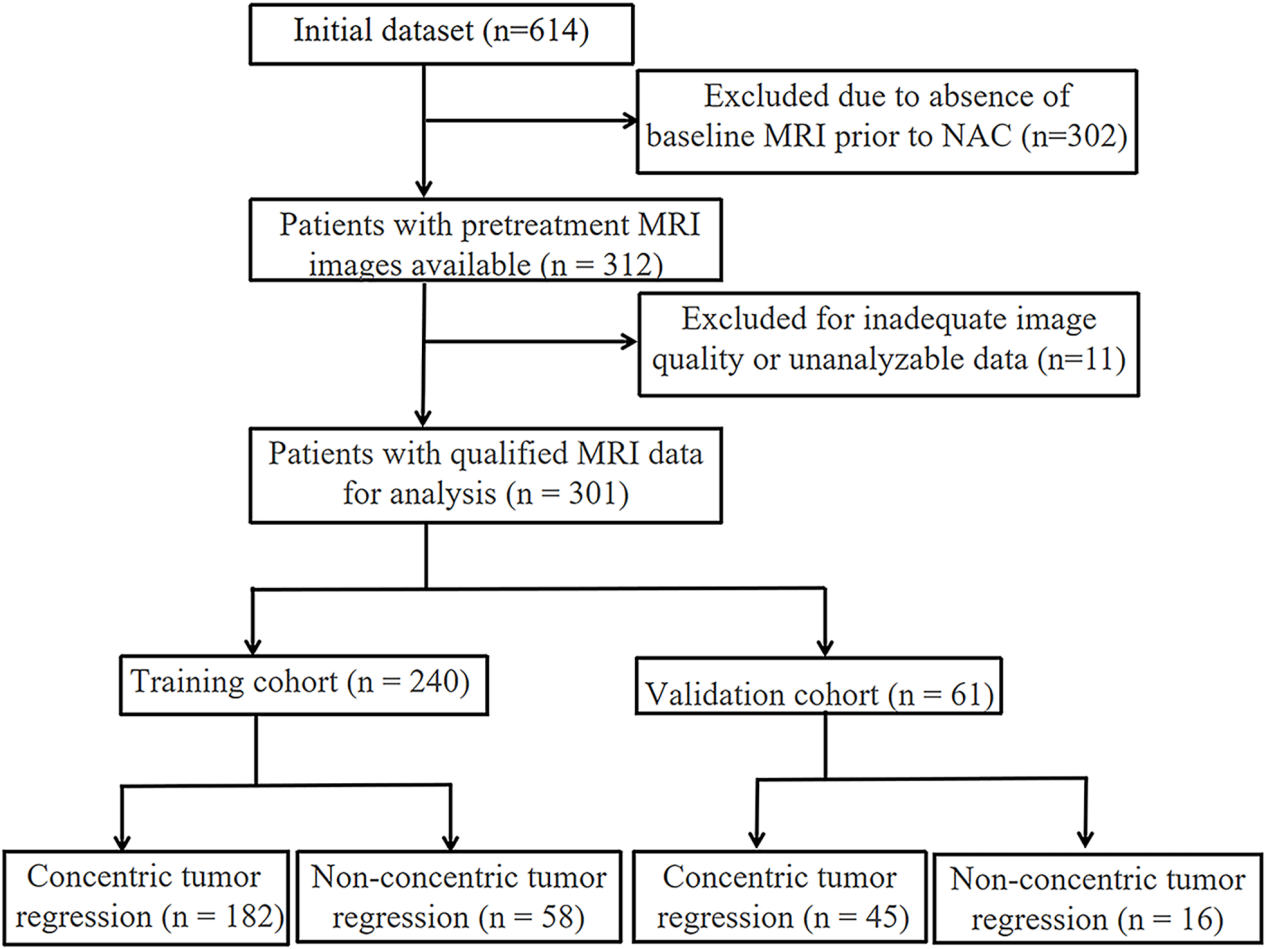
Flowchart of patient inclusion in the study.

The participant screening workflow is depicted in Figure 1, and a summary of the main clinical variables for both training and validation sets is provided in Table 1. A total of 240 individuals comprised the training dataset, and 61 patients were allocated to the testing group. Based on post-NAC tumor regression profiles, all cases were categorized into either the CR or NCR subtypes.

**Table 1:** Comparison of clinical characteristics between the training and validation cohorts.

Characteristic	Development set				Validation set			
	ALL	NCR	CR	*p*	ALL	NCR	CR	*p*
	(*n* = 240)	(*n* = 58)	(*n* = 182)	value	(*n* = 61)	(*n* = 16)	(*n* = 45)	value
Age (years) (Mean ± std)	51.80 ± 9.62	49.55 ± 10.12	52.52 ± 9.38	0.09	51.20 ± 8.73	51.88 ± 9.70	50.96 ± 8.46	0.72
ER (Mean ± std)	48.31 ± 39.50	63.91 ± 34.60	43.34 ± 39.75	0.00	46.72 ± 39.64	51.88 ± 38.85	44.89 ± 40.19	0.49
PR (Mean ± std)	33.81 ± 36.75	39.76 ± 34.97	31.92 ± 37.19	0.06	30.85 ± 36.06	38.44 ± 39.32	28.16 ± 34.90	0.74
Ki67 (Mean ± std)	38.09 ± 20.34	35.95 ± 17.28	38.77 ± 21.22	0.51	38.98 ± 22.58	33.62 ± 21.67	40.89 ± 22.82	0.32
Molecular Phenotype (Mean ± std)	3.43 ± 1.26	3.27 ± 1.27	3.48 ± 1.26	0.37	3.33 ± 1.40	3.19 ± 1.52	3.39 ± 1.37	0.61
Menopausal status				0.67				0.71
Postmenopausal	112 (46.67)	29 (50.00)	83 (45.60)		30 (49.18)	9 (56.25)	21 (46.67)	
Positive	128 (53.33)	29 (50.00)	99 (54.40)		31 (50.82)	7 (43.75)	24 (53.33)	
FISH				0.41				0.35
Negative	144 (60.00)	38 (65.52)	106 (58.24)		34 (55.74)	11 (68.75)	23 (51.11)	
Positive	96 (40.00)	20 (34.48)	76 (41.76)		27 (44.26)	5 (31.25)	22 (48.89)	
Clinical T stage				0.12				0.19
Clinical N stage				0.09				0.34
0	43 (17.92)	7 (12.07)	36 (19.78)		8 (13.11)	null	8 (17.78)	
1	162 (67.50)	37 (63.79)	125 (68.68)		42 (68.85)	13 (81.25)	29 (64.44)	
2	17 (7.08)	7 (12.07)	10 (5.49)		4 (6.56)	1 (6.25)	3 (6.67)	
3	18 (7.50)	7 (12.07)	11 (6.04)		7 (11.48)	2 (12.50)	5 (11.11)	

Among training set participants, the mean age for the CR subgroup was 52.52 ± 9.38 years, slightly exceeding the NCR group average of 49.55 ± 10.12 years; however, this age difference was not statistically significant (*p* = 0.086). No significant intergroup difference was observed in Ki-67 expression levels (38.77 ± 21.22 vs. 35.95 ± 17.28, *p* = 0.514), HER2 status as determined by FISH (*p* = 0.406), or clinical stage classification (*p* = 0.116).

A similar pattern was evident within the test set, where baseline metrics also showed no significant distinction between CR and NCR groups (all *p* > 0.05). The homogeneity of clinical characteristics between the two datasets provided a reliable base for subsequent radiomic model development.

### 3.2 Development and validation of clinicopathological signature

#### 3.2.1 Model comparison

In the training set, univariate analysis revealed several factors that may be associated with tumor regression patterns following NAC in breast cancer patients. These factors included age, clinical N stage, and the expression levels of ER and PR. Machine learning models were then trained using these clinicopathological variables.

Among all models tested, Random Forest demonstrated the highest performance on the training set, achieving an accuracy of 92.5% and an exceptional AUC of 0.99. This model exhibited excellent sensitivity (90.1%) and specificity (100%), making it highly effective in identifying true positives and true negatives. However, on the test set, its performance decreased, with an accuracy of 57.4% and an AUC of 0.57. The sensitivity of 60% and PPV of 77.1% suggest that while the model was highly accurate in training, its ability to generalize to unseen data was limited.

#### 3.2.2 Performance metrics

**Figure 2:**
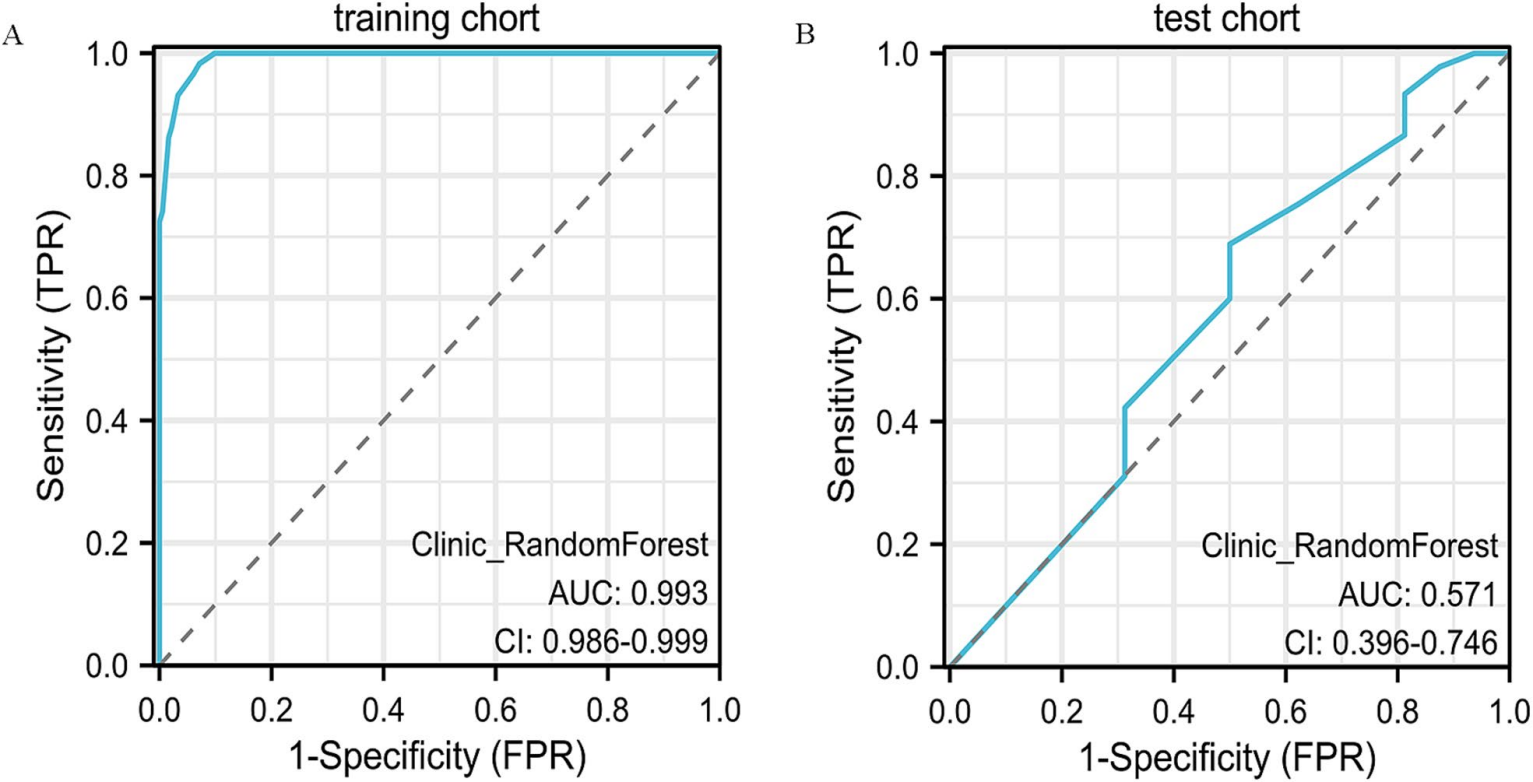
ROC curve analysis of the clinical model. **(A)** ROC curve of the clinical model in the training cohort, showing its discrimination between different outcome groups. **(B)** ROC curve in the validation cohort, confirming the model’s predictive accuracy and generalizability.

Models such as LightGBM, LR, k-nearest neighbors (KNN), SVM, and multilayer perceptron (MLP) exhibited more mixed results. LightGBM, for example, achieved an accuracy of 39.3% on the test set, with a very low sensitivity (17.8%) and high specificity (100%), but its overall predictive capability was limited. MLP and LR also faced similar challenges in terms of generalizability, with performance drops in test data, especially in terms of sensitivity and specificity. The ROC curves of the RF model are presented (Figures 2A,B), with additional comparative metrics summarized (Table 2).

**Table 2:** Comparison of the clinical–pathological model performance based on AUC, accuracy, and other evaluation metrics.

Clinical model		Accuracy	AUC	95% CI	Sensitivity	Specificity	PPV	NPV
LR	Train	0.67	0.70	0.6272–0.7779	0.67	0.66	0.86	0.39
	test	0.57	0.54	0.3784–0.7063	0.56	0.63	0.81	0.33
SVM	Train	0.70	0.70	0.6201–0.7873	0.72	0.66	0.87	0.43
	test	0.67	0.41	0.2236–0.5861	0.84	0.19	0.75	0.30
KNN	Train	0.51	0.81	0.7560–0.8634	0.35	1.00	1.00	0.33
	test	0.71	0.55	0.3782–0.7134	0.87	0.25	0.77	0.40
RandomForest	Train	0.93	0.99	0.9862–0.9991	0.90	1.00	1.00	0.76
	test	0.57	0.57	0.3957–0.7460	0.60	0.50	0.77	0.31
ExtraTrees	Train	0.24	1.00	0.9987–1.0000	0.00	1.00	0.00	0.24
	test	0.71	0.54	0.3726–0.7136	0.89	0.19	0.76	0.38
XGBoost	Train	0.84	0.92	0.8762–0.9590	0.83	0.86	0.95	0.62
	test	0.72	0.56	0.3937–0.7272	0.89	0.25	0.77	0.44
LightGBM	Train	0.67	0.80	0.7347–0.8639	0.61	0.85	0.93	0.41
	test	0.39	0.56	0.3931–0.7235	0.18	1.00	1.00	0.30
MLP	Train	0.71	0.74	0.6651–0.8171	0.71	0.71	0.88	0.44
	test	0.71	0.44	0.2631–0.6188	0.91	0.13	0.75	0.33

### 3.3 Construction and validation of radiomic model

#### 3.3.1 Feature selection

**Figure 3:**
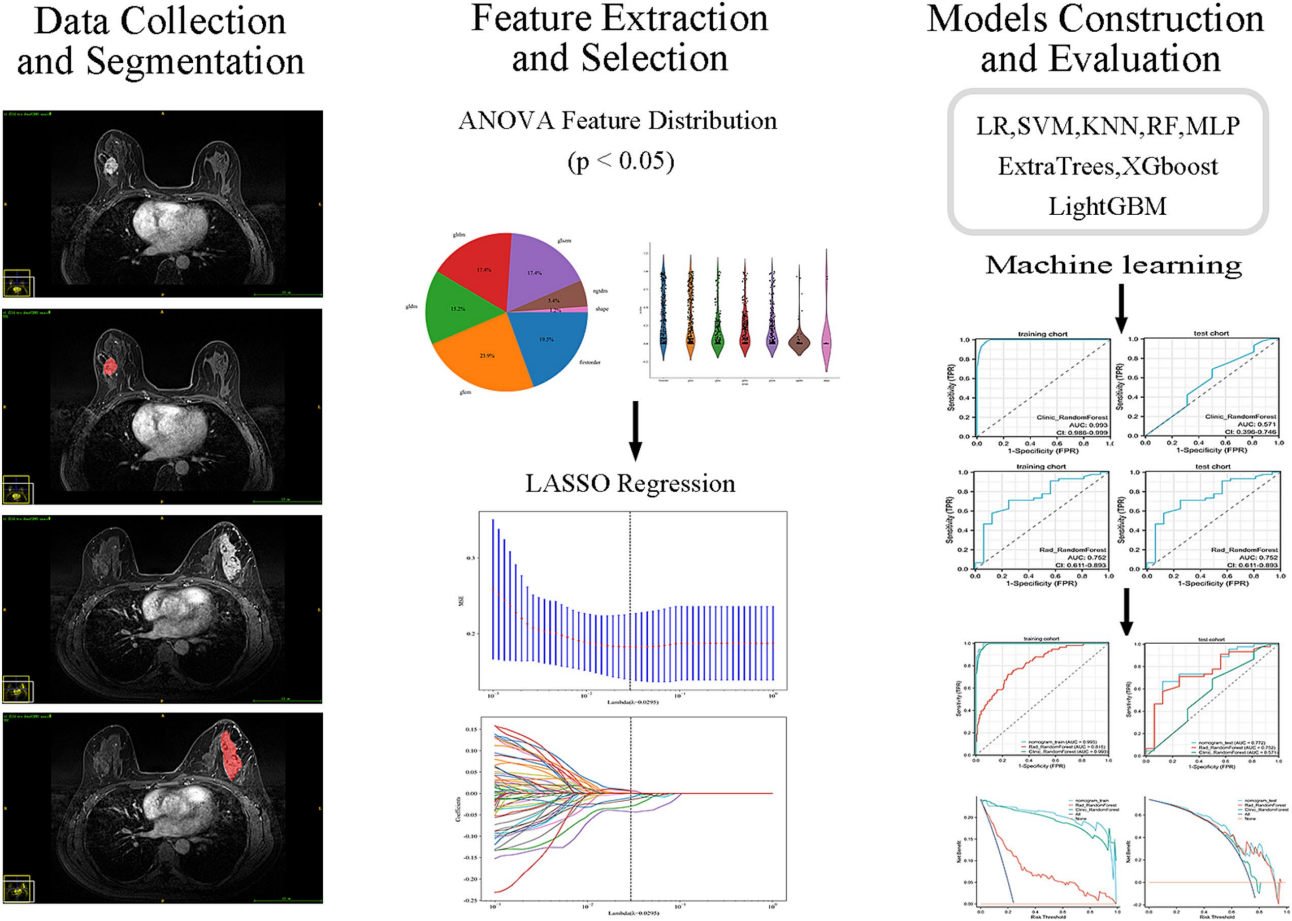
Workflow of MRI-based radiomics model development. Flowchart illustrating the main steps of model development, including MRI acquisition, tumor segmentation, radiomic feature extraction, feature selection, model construction, and validation.

**Figure 4:**
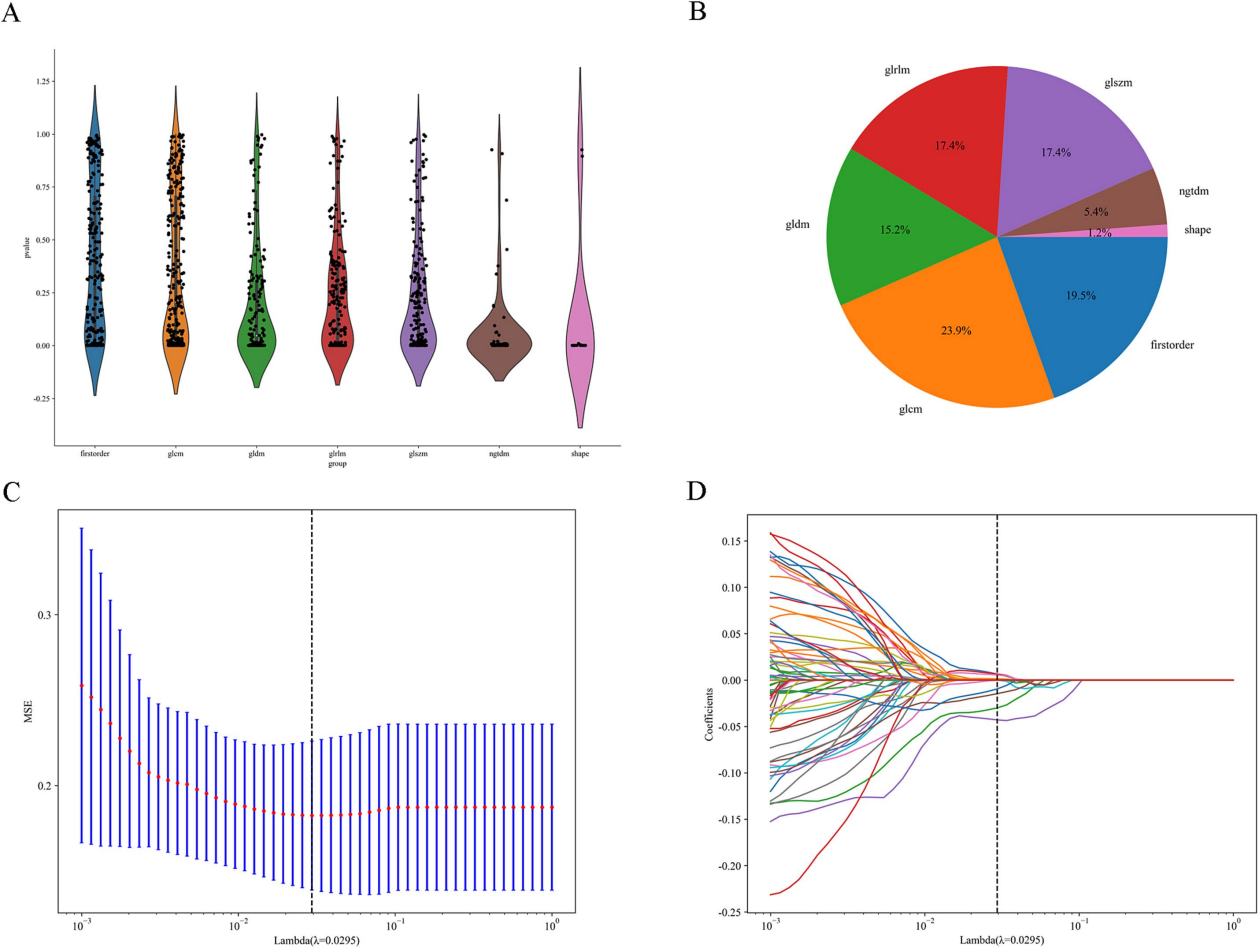
Feature selection process. **(A)** Distribution of the filtered features. **(B)** Proportion of features selected by each filtering method. **(C,D)** LASSO regression plots.

Figure 3 shows the process of constructing machine learning models with radiomic features, clinicopathological data, and nomograms. MRI images of 301 patients were manually segmented layer by layer, followed by feature extraction. A total of 1,196 features were extracted from pre-treatment images. Through one-way analysis of variance, 498 significant features were selected, and the Pearson correlation coefficient was calculated. Features with a correlation higher than 0.9 were reduced by keeping only one. Finally, 84 features were further selected using lasso regression, which identified 8 features that were most relevant to predicting the tumor regression patterns (Figure 4).

#### 3.3.2 Model comparison

We evaluated the performance of various machine learning models in predicting tumor regression patterns following NAC in breast cancer patients. Among the models evaluated, XGBoost demonstrated the best performance on the training set, achieving an impressive accuracy of 87.5% and an AUC of 0.95, with a sensitivity of 86.3% and specificity of 91.4%. This model outperformed others in terms of both predictive accuracy and the ability to distinguish between responders and non-responders to neoadjuvant therapy. However, on the test set, its performance slightly decreased, with an accuracy of 57.4% and an AUC of 0.65. Despite this drop, it still maintained a relatively high PPV of 91.3%, indicating its effectiveness in predicting positive regression outcomes. Random Forest also showed strong performance, with an accuracy of 74.6% and an AUC of 0.82 on the training set. On the test set, it had an accuracy of 70.5%, with an AUC of 0.75, and a sensitivity of 68.9%. This model demonstrated a good balance between sensitivity and specificity, making it a robust choice for identifying tumor regression.

#### 3.3.3 Performance metrics

**Figure 5:**
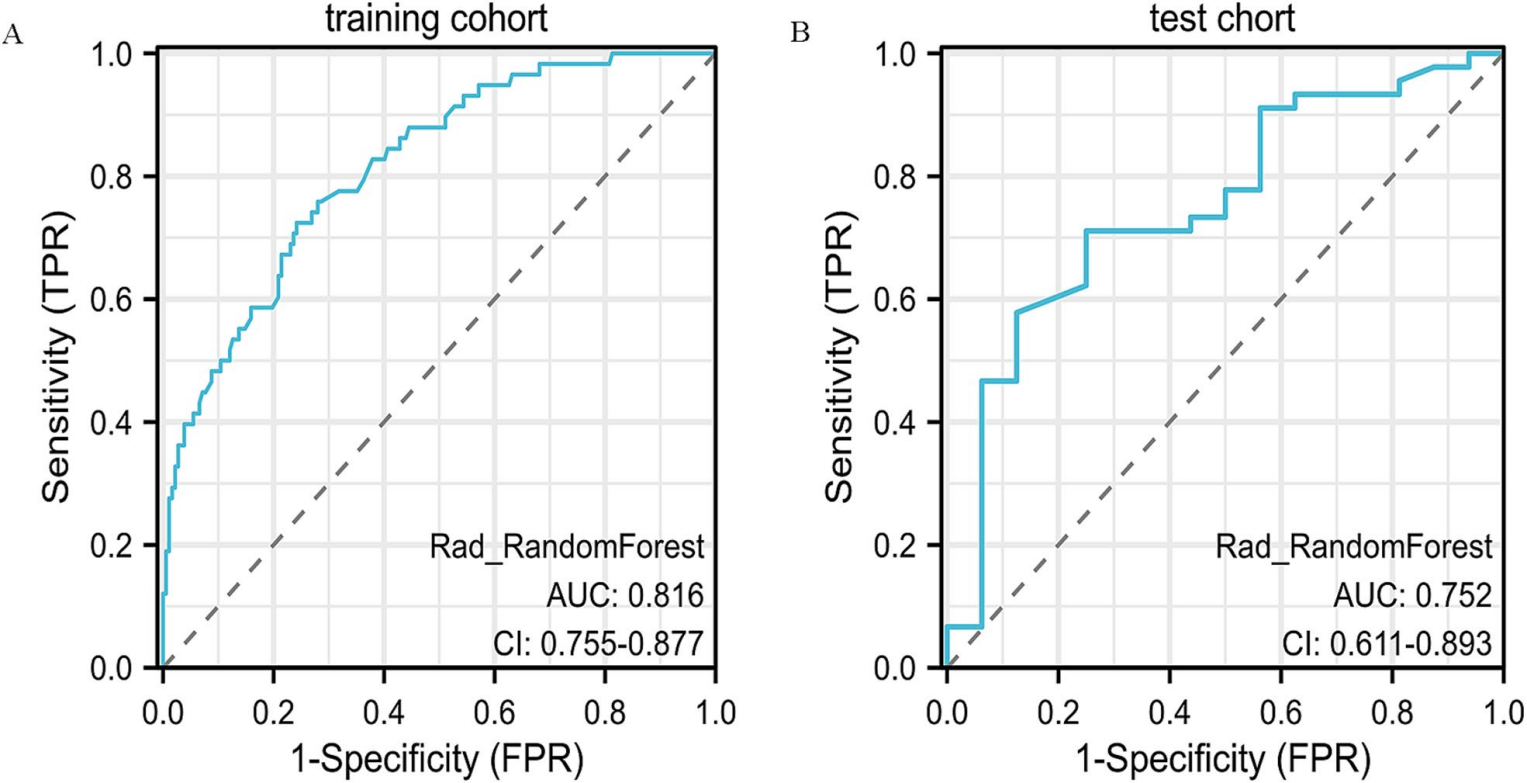
ROC curve analysis of the radiomics model. **(A)** ROC curve for the training dataset, demonstrating the predictive performance of the radiomics model. **(B)** ROC curve for the validation dataset, showing the model’s stability and reproducibility in an independent cohort.

Other models such as SVM, LightGBM, and MLP also provided reasonable performance, but none exceeded the performance of XGBoost or Random Forest in terms of AUC or accuracy. SVM, for example, achieved an AUC of 0.806 on the training set, but its test set performance was lower, with an accuracy of 55.7% and AUC of 0.68. In conclusion, Random Forest provided a good balance of predictive accuracy and clinical applicability, especially on the test set. The ROC curves of the RF model are presented (Figures 5A,B), with additional comparative metrics summarized (Table 3). To provide a concise overview of the radiomics workflow, including feature selection and model evaluation, an additional summary table was compiled. The sequential feature screening steps and comparative performance of all applied machine learning algorithms are summarized in Table 4.

**Table 3:** Comparison of the radiomics model performance based on AUC, accuracy, and other evaluation metrics.

Radiomic model		Accuracy	AUC	95% CI	Sensitivity	Specificity	PPV	NPV
LR	Train	0.55	0.67	0.5954–0.7468	0.45	0.88	0.92	0.34
	test	0.72	0.75	0.6085–0.8887	0.69	0.81	0.91	0.48
SVM	Train	0.82	0.81	0.7282–0.8842	0.84	0.78	0.92	0.60
	test	0.56	0.68	0.5235–0.8265	0.47	0.81	0.88	0.35
KNN	Train	0.53	0.79	0.7314–0.8430	0.39	1.00	1.00	0.34
	test	0.57	0.61	0.4428–0.7711	0.58	0.56	0.79	0.32
RandomForest	Train	0.75	0.82	0.7550–0.8768	0.75	0.72	0.90	0.48
	test	0.71	0.75	0.6111–0.8931	0.69	0.75	0.89	0.46
ExtraTrees	Train	0.62	0.72	0.6493–0.7987	0.57	0.76	0.88	0.36
	test	0.56	0.65	0.4962–0.8121	0.47	0.81	0.88	0.35
XGBoost	Train	0.88	0.95	0.9285–0.9787	0.86	0.91	0.97	0.68
	test	0.57	0.65	0.4999–0.8029	0.47	0.88	0.91	0.37
LightGBM	Train	0.80	0.87	0.8165–0.9150	0.82	0.74	0.91	0.57
	test	0.69	0.69	0.5425–0.8325	0.67	0.75	0.88	0.44
MLP	Train	0.57	0.69	0.6126–0.7639	0.50	0.81	0.89	0.34
	test	0.69	0.72	0.5729–0.8688	0.64	0.81	0.91	0.45

**Table 4:** Summary of the feature selection workflow and model performance.

Step	Method/Model	Purpose	Number of Features	AUC (Training)	AUC (Validation)
1	Univariate analysis	Identification of statistically significant features associated with tumor regression (*p* < 0.05).	1,196 → 498	-	-
2	Pearson correlation	Removal of redundant variables (*r* > 0.9).	498 → 84	-	-
3	LASSO regression	Selection of the most predictive and non-collinear features.	84 → 8	-	-
4	Random Forest	Model training using the selected features.	8	0.82	0.75
5	XGBoost	Gradient boosting framework tested for predictive accuracy and robustness.	8	0.95	0.65
6	SVM	Comparative model testing using kernel-based classification.	8	0.81	0.68
7	LightGBM	Evaluation of gradient boosting model to test feature robustness and generalization.	8	0.87	0.69
8	MLP	Assessment of multilayer perceptron neural network for nonlinear pattern recognition.	8	0.69	0.72
9	LR	Baseline linear classifier used for model comparison and interpretability.	8	0.70	0.54

### 3.4 Nomogram development and validation

#### 3.4.1 Nomogram construction

**Figure 6:**
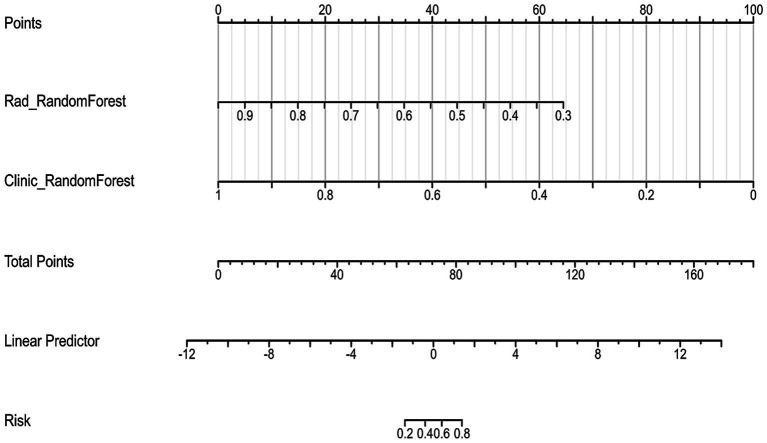
Nomogram for predicting tumor regression patterns after neoadjuvant chemotherapy (NAC).

To create a more reliable prediction tool for assessing tumor regression patterns following NAC in breast cancer patients, we developed a nomogram that integrates the top-performing machine learning models based on both clinicopathological and radiomic signatures (Figure 6). Notably, the Random Forest model emerged as the best performer for both clinical and radiomic feature-based signatures, making it the ideal choice for inclusion in the nomogram.

#### 3.4.2 Nomogram validation

**Figure 7:**
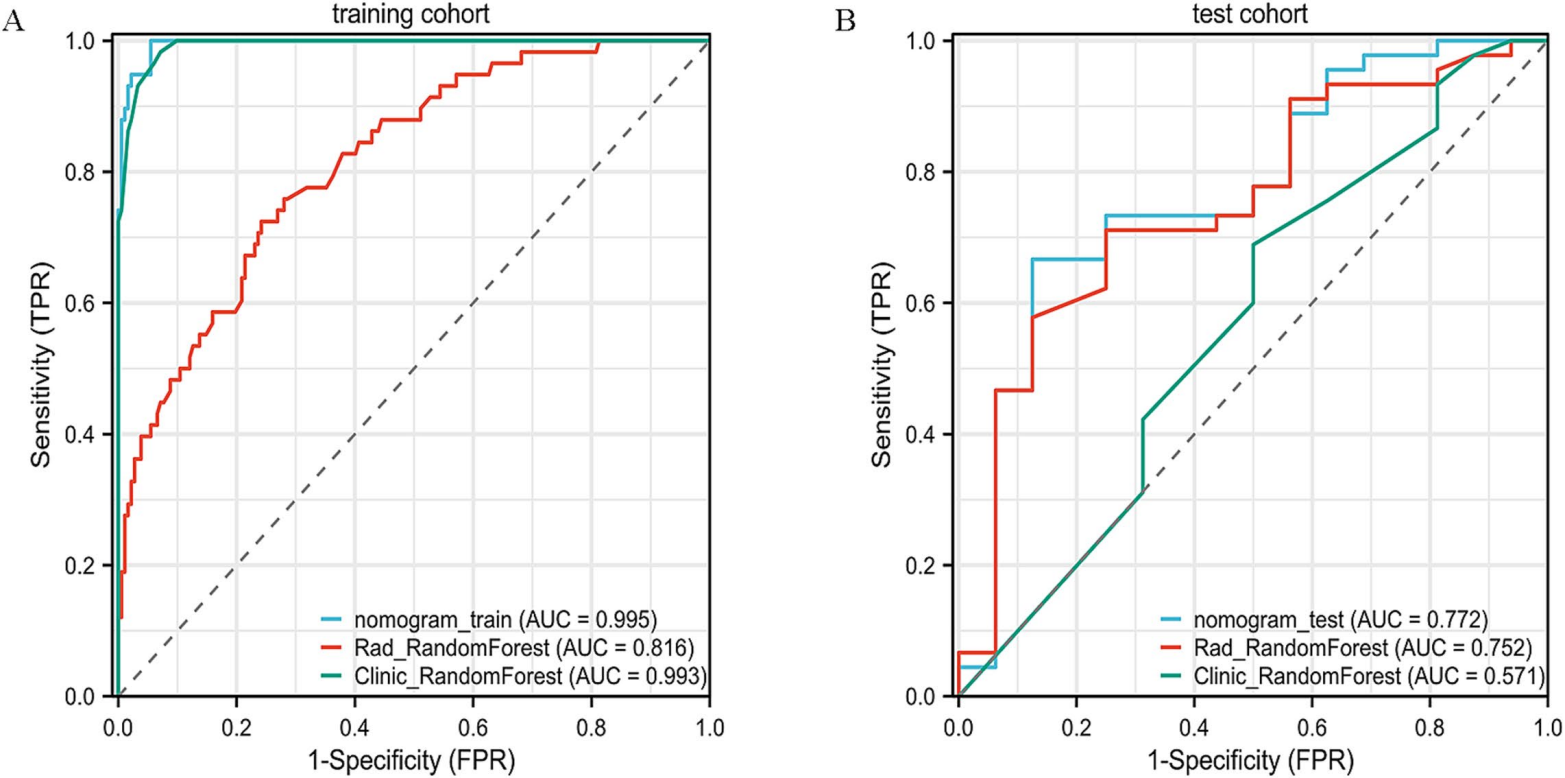
ROC curve analysis of the nomogram. **(A)** Training cohort. **(B)** Validation cohort.

When evaluated on the training set, the nomogram achieved an impressive AUC of 0.99, with a sensitivity of 0.95 and specificity of 0.96 (Figure 7A). The PPV on the training set was 0.85, demonstrating the model’s strong predictive capacity.

**Figure 8:**
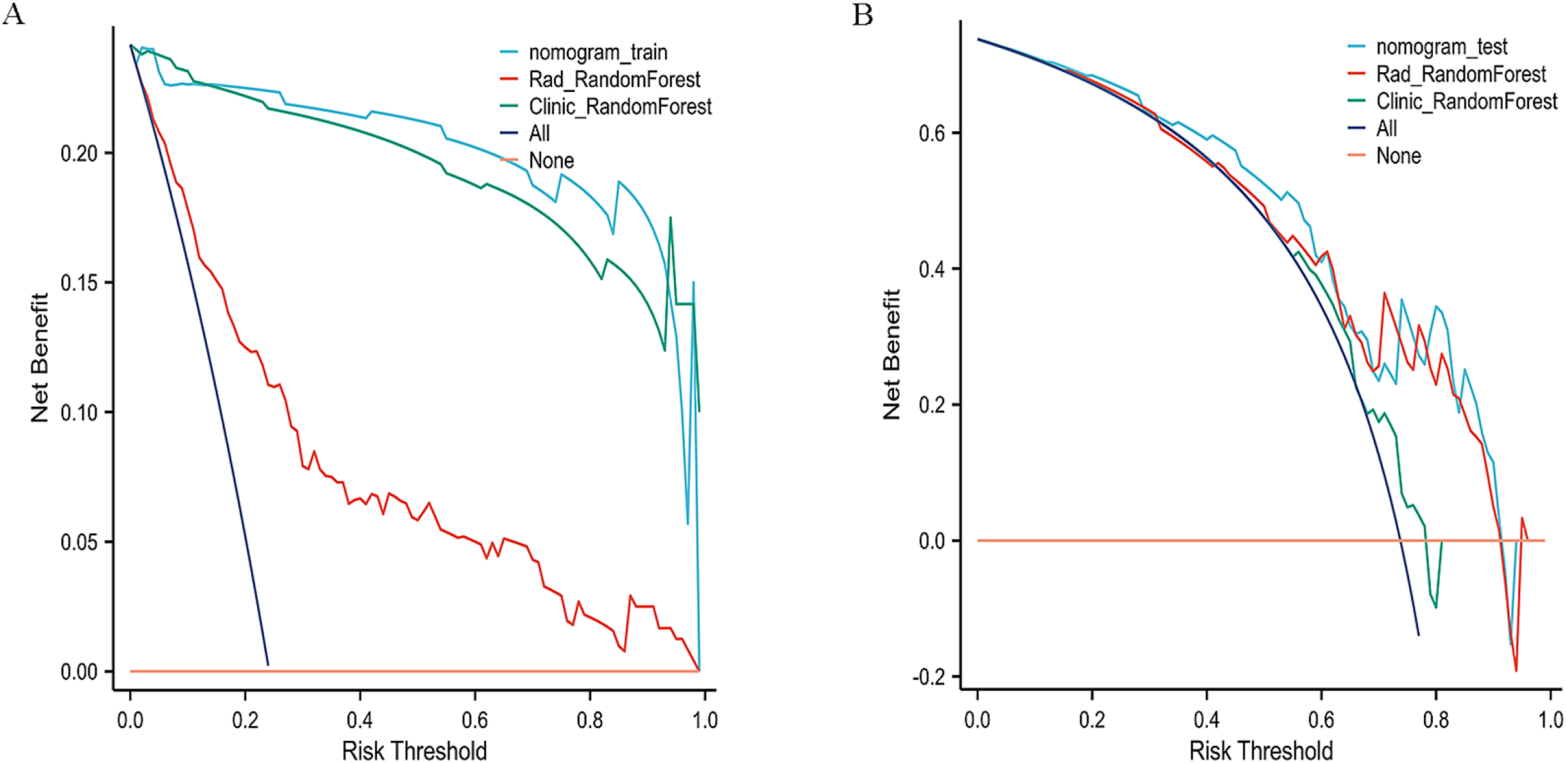
Decision curve analysis (DCA) of the nomogram. **(A)** Training cohort. **(B)** Validation cohort.

On the test set, the nomogram performed well, with an accuracy of 0.70 and an AUC of 0.75, as illustrated by the ROC curve (Figure 7B). It achieved a sensitivity of 0.64 and specificity of 0.88, indicating its ability to effectively identify both responders and non-responders to NAC. Furthermore, the PPV was 0.94, and the NPV was 0.46, reflecting the nomogram’s good predictive reliability for clinical decision-making. Decision Curve Analysis (DCA) demonstrated that the combined model provided greater net benefit than the radiomics and clinical models in both the training and validation cohorts (Figures 8A,B).

To further validate the nomogram, its discrimination and clinical utility were assessed in both cohorts. The ROC and decision curve analyses demonstrated consistent predictive performance and good agreement between predicted and observed outcomes. Compared with the Random Forest (AUC = 0.75) and XGBoost (AUC = 0.65) models, the nomogram showed superior discrimination and higher clinical usefulness, providing stronger support for preoperative decision-making.

Beyond the primary accuracy outcomes, 95% confidence intervals were also estimated for AUC values to reinforce the reliability of the statistical findings. Comparative results revealed that the nomogram achieved marginally greater consistency and predictive steadiness than either the Random Forest or XGBoost models across datasets. The comparative performance of the clinical, radiomic, and integrated models is summarized in Table 5, highlighting the improved predictive consistency achieved by combining imaging and clinical features.

**Table 5:** Overview of predictive performance among the top-performing clinical, radiomic, and nomogram models.

Model type	Best algorithm	AUC (Training)	AUC (Validation)	Accuracy	Sensitivity	Specificity
Clinical model	Random Forest	0.99	0.57	0.57	0.60	0.50
Radiomic model	Random Forest	0.82	0.75	0.71	0.69	0.75
Nomogram	Random Forest + Clinical features	0.99	0.75	0.70	0.64	0.88

## 4 Discussion

In this work, we developed a nomogram that incorporates radiomic parameters from pre-NAC T1-weighted MRI alongside selected clinicopathological variables to classify tumor regression patterns—namely, CR and NCR responses—in breast cancer. The model yielded high classification efficacy, with AUC values of 0.99 and 0.75 in the training and external test sets, respectively, demonstrating its reliability and adaptability to different patient populations. The relatively large cohort size enhanced the statistical robustness and strengthened the clinical generalizability of the findings. Feature extraction was carried out using a rigorously controlled procedure, which included expert-guided segmentation of baseline MRI scans, followed by multi-step filtering with ANOVA, Pearson correlation, and LASSO-based selection. This comprehensive pipeline ensured methodological consistency and reproducibility. By fusing quantitative imaging traits with pathological profiles, the resulting nomogram supports anticipatory surgical planning and individualized therapy selection.

Recognizing the spatial variability in tumor response after NAC, we examined the predictive capacity of clinicopathological indicators in breast cancer. Univariate statistical testing (*p* < 0.1) revealed estrogen receptor (ER) positivity (*p* = 0.002) and cN stage (*p* = 0.093) as significant correlates, aligning with the biological principles of the RCB system (18). Notably, ER-positive cases were more frequently associated with non-concentric regression (63.91 ± 34.60%) compared to ER-negative tumors (43.34 ± 39.75%, *p* = 0.002), suggesting a potential connection between hormonal activity and chemotherapy response (19). To assess model efficacy, several supervised classification algorithms were applied, including Random Forest and XGBoost. In the training dataset, Random Forest achieved the highest accuracy (AUC = 0.993, 95% CI: 0.986–0.999), outperforming logistic regression (AUC = 0.70). However, in the independent test cohort, its predictive power diminished (AUC = 0.57), reflecting the limitations of models based exclusively on clinical variables. This evident gap between training and validation performance indicates that the Random Forest model may have partially overfitted the training data. Such behavior is frequently observed when the number of predictors outweighs the sample size, causing the model to learn cohort-specific variations rather than generalizable patterns. Introducing stricter feature filtering, cross-validation, and careful parameter tuning could improve the model’s robustness and consistency when applied to independent datasets. These results are consistent with Bitencourt et al. (20), who reported improved diagnostic accuracy in HER2-positive patients using integrated radiomic-clinical frameworks (AUC = 0.89), compared to clinical-only approaches (AUC = 0.61). Moreover, the significance of cN staging echoes conclusions from a multicenter investigation by Yu et al., which demonstrated the association between nodal involvement and NAC response (21). While age (*p* = 0.086) and progesterone receptor (PR) status (*p* = 0.06) were not statistically significant, older patients exhibited a higher incidence of CR (52.52 ± 9.38 vs. 49.55 ± 10.12 years), potentially reflecting immunological differences with age that warrant further research.

To build a robust model for predicting tumor regression subtypes following NAC, we employed a range of supervised learning methods, including LR, SVM, RF, and XGBoost. These algorithms were selected due to their established performance in radiomics-based cancer prediction tasks (18, 20, 21). To mitigate overfitting, a two-stage variable reduction strategy was adopted: initial univariate analysis (*p* < 0.05) and Pearson correlation filtering (r > 0.9) were used to eliminate redundant features, followed by LASSO regression to retain the most predictive variables. Out of 1,196 extracted radiomic features, 84 independent parameters remained, from which the top 8 were incorporated into the final classifier. Among the models tested, RF delivered the most consistent predictive ability, with AUCs of 0.816 and 0.75 in the training and validation sets, respectively. In contrast, although XGBoost performed well in the training group (AUC = 0.95), it demonstrated reduced generalization capacity in the validation data (AUC = 0.65), suggesting overfitting—a recognized challenge in radiomics applications involving high-dimensional inputs (20, 21). Likewise, the noticeable reduction in XGBoost performance from training to validation cohorts reinforces the need for stronger regularization and systematic hyperparameter optimization. Implementing nested cross-validation, adjusting learning rates, or constraining tree depth may further mitigate model variance and enhance predictive reliability across unseen data. These results underscore the need for larger datasets and algorithm refinement to enhance external validity and clinical applicability.

In this study, radiomic features were extracted from various domains, such as first-order statistical measures, texture descriptors like the gray-level co-occurrence matrix (GLCM), and morphological characteristics, including sphericity and surface area. Among these, “original-shape-Sphericity” and “wavelet-LLH-ngtdm-Busyness” exhibited the highest ability to distinguish between tumor regression patterns, emphasizing the relevance of tumor shape and internal heterogeneity in defining CR and NCR (13, 22, 23). This finding aligns with previous studies, such as those by Li et al., who highlighted the importance of morphological features in evaluating treatment response (14). In addition, Braman et al. demonstrated the value of texture-based metrics in reflecting tumor microenvironment complexity (23). One of the strengths of our model is the integration of eight radiomic features—selected through LASSO regularization—with four key clinical variables (age, ER, PR, and cN stage). This composite model achieved an AUC of 0.75 in the validation cohort, with sensitivity and specificity values of 0.64 and 0.88, respectively. Importantly, the specificity of the combined model was significantly higher than the clinical-only model (0.88 vs. 0.50), which underscores its potential for guiding clinical decisions. When predicting centripetal regression, the model achieved a positive predictive value of 94%, demonstrating its practical value in preoperative planning, especially for decisions regarding breast conservation. These results are consistent with findings from Yu et al. and Bitencourt et al., both of whom reported improved predictive accuracy when combining radiomic and clinical features (20, 21). Additionally, the correlation between ER-negative status or advanced cN stage with non-concentric regression is in line with previous studies that identified these factors as associated with poorer chemotherapy responses (19, 24). The nomogram based on this integrated model offers a straightforward and clinically relevant tool for assessing individual patient risk, aiding in personalized treatment planning.

To further ensure clinical translatability, the predictive framework should be validated on larger, independent, and multi-institutional cohorts. Expanding the dataset and standardizing MRI acquisition parameters will help reduce bias and confirm the model’s stability under varying imaging conditions, thereby improving its generalizability for real-world applications.

Although the combined model demonstrated favorable predictive capability, enhancing statistical clarity and validating its performance with external cohorts remain important. Incorporating calibration assessment and interval estimation could improve interpretability. The nomogram performed steadily compared with single-model approaches, yet confirmation through multicenter data is required to ensure broader applicability.

This study has several limitations. Due to its retrospective design at a single institution, there is a potential risk of selection bias, which may affect the external applicability of the results. The relatively small size of the validation cohort (*n* = 61) further limits the statistical power and generalizability of the findings. To validate and extend these observations, future research should incorporate a multicenter, prospective design, such as the I-SPY2 framework, which will allow for more robust conclusions across diverse patient populations (25). Additionally, incorporating complementary imaging modalities like dynamic contrast-enhanced MRI (DCE-MRI), apparent diffusion coefficient (ADC) mapping, and T2-weighted imaging (T2WI) could improve the model’s predictive power by offering a more comprehensive view of tumor biology.

In conclusion, the MRI-based clinical–radiomic fusion model developed in this study successfully stratified tumor regression patterns in breast cancer patients undergoing NAC. By integrating both imaging features and clinical data, the model provides a practical decision-support tool for personalized treatment planning, particularly for breast-conserving surgeries. Future efforts should focus on validating the model in larger, more diverse cohorts and exploring the integration of genomic or molecular biomarkers to further enhance its clinical relevance and translational potential.

## 5 Conclusion

The integration of imaging biomarkers and clinical profiles enables reliable prediction of tumor response post-NAC, supporting more informed and tailored treatment strategies.

## Data Availability

The original contributions presented in the study are included in the article/supplementary material, further inquiries can be directed to the corresponding authors.
